# Resource Limitation, Controphic Ostracod Density and Larval Mosquito Development

**DOI:** 10.1371/journal.pone.0142472

**Published:** 2015-11-11

**Authors:** Raylea Rowbottom, Scott Carver, Leon A. Barmuta, Philip Weinstein, Dahlia Foo, Geoff R. Allen

**Affiliations:** 1 School of Land and Food/TIA, University of Tasmania, Hobart, Australia; 2 School of Biological Sciences, University of Tasmania, Hobart, Australia; 3 School of Pharmacy and Medical Science, University of South Australia, Adelaide, Australia; Swedish University of Agricultural Sciences, SWEDEN

## Abstract

Aquatic environments can be restricted with the amount of available food resources especially with changes to both abiotic and biotic conditions. Mosquito larvae, in particular, are sensitive to changes in food resources. Resource limitation through inter-, and intra-specific competition among mosquitoes are known to affect both their development and survival. However, much less is understood about the effects of non-culicid controphic competitors (species that share the same trophic level). To address this knowledge gap, we investigated and compared mosquito larval development, survival and adult size in two experiments, one with different densities of non-culicid controphic conditions and the other with altered resource conditions. We used *Aedes camptorhynchus*, a salt marsh breeding mosquito and a prominent vector for Ross River virus in Australia. *Aedes camptorhynchus* usually has few competitors due to its halo-tolerance and distribution in salt marshes. However, sympatric ostracod micro-crustaceans often co-occur within these salt marshes and can be found in dense populations, with field evidence suggesting exploitative competition for resources. Our experiments demonstrate resource limiting conditions caused significant increases in mosquito developmental times, decreased adult survival and decreased adult size. Overall, non-culicid exploitation experiments showed little effect on larval development and survival, but similar effects on adult size. We suggest that the alterations of adult traits owing to non-culicid controphic competition has potential to extend to vector-borne disease transmission.

## Introduction

The effectiveness of exploitative competition for available food resources is driven by the presence of species within the same trophic level (controphic species) and species that share the same resources and functional feeding group [1, 2]. For mosquitoes, the presence of other species that are filter- and suspension-feeders can limit the amount of available food [3]. Consequently, the outcomes of controphic resource (exploitative) competition on mosquito development and survival should be equivalent to increased intra-specific resource limitation; with greater effects on mosquito development, survival and adult size with increasing competition. However, this hypothesis is yet to be investigated.

Over the last two decades, significant effort has been directed to understanding the diverse effects that biotic interactions have on the ecology of mosquito vectors [1, 4–7]. The larval life-stages of mosquitoes are the most sensitive to biotic interactions with impacts on developmental times, survival or changes to adult size. These impacts can, in turn, effect fecundity and, for vectors of disease, vector competence capacity [8–10]. To date, investigations have largely focused on larval predation, inter- and intra- specific mosquito competition (particularly with *Aedes aegypti* (Linnaeus) and *Aedes albopictus* (Say)) and mosquito oviposition behaviour as a means of predation and competition avoidance [11, 12]. The importance of competitive interactions with other non-culicid invertebrates, particularly non-culicid controphic species is poorly understood, yet may play an important role in mosquito abundance within natural conditions, with consequent implications for vectors [13, 14].

Across southern Australia *Ae*. *camptorhynchus* (Thomson) [15, 16] is a major vector of Ross River virus (RRV: Togaviridae: *Alphavirus*). Epidemiologically, RRV is Australia’s most important vector-borne disease with clinical notifications ranging between 1451 – 7754 per annum, resulting in an annual economic impact of approximately $15 million dollars [17]. Salt marshes are particularly important habitats for this halo-tolerant vector due to the hyper-saline aquatic conditions. Such physiologically extreme environments result in lower aquatic species richness, and hence fewer predators and competitors of *Ae*. *camptorhynchus*, which is indicative of regions where this mosquito occurs across Australia [15, 18–20]. In these habitats *Ae*. *camptorhynchus* lays desiccation resistant eggs that undergo mass hatching with large pulses of rainfall or tidal inundations [21, 22]. Likewise, these pulses of water also result in high densities of micro-crustaceans that are capable of surviving dry periods [23, 24]. The *Diacypris* spp. (Crustacea: Ostracoda) that dominate the remaining aquatic fauna in these environments [25] are detritovores/herbivores, occupying the same functional feeding group as *Ae*. *camptorhynchus*. In addition, a negative density relationship from field evidence suggests these species may interact via exploitative competition [26]. If exploitative competition is occurring between these species we expect it will impact mosquito vector development, survival, abundance and size [26].

In this study we test the hypothesis that non-culicid controphic exploitative competition with ostracods reflects intra-specific resource limitation. The reciprocal of our hypothesis is that exploitative competition is not occurring between these taxa and ostracods will therefore have no impact on *Ae*. *camptorhynchus* development and survival. We examined our hypothesis using two experiments: an intra-specific resource limitation experiment with low densities of *Ae*. *camptorhynchus*; and a non-culicid controphic exploitative competition experiment with ostracods in increasing densities. Between the two experiments we contrast the changes in larval developmental times and survival, and effects on adult size. For both experiments, we predict decreased larval survival, increased development time, and reduced adult size as resources become limiting and controphic competition increases. We observed development and survival changes with intra-specific resource limitation and changes in adult size for both intra-specific resource limitation and controphic competition treatments. Our findings suggest no evidence of exploitative competition among *Ae*. *camptorhynchus* and ostracods during larval development, but detectable similarities to resource limitation with adult size.

## Materials and Methods

### Invertebrate collections

All mosquitoes and ostracods used in this study were sourced from the Primrose Sands salt marsh (147°39 East, 42°52 South), east of Hobart, Tasmania, whereby permission to access the marsh was granted by the land owner. Water bodies found in the Primrose Sands salt marsh are very ephemeral, lasting on average only 14 days after inundation over the peak of summer [27]. Such environments are very depauperate of aquatic species diversity as shown by Carver *et*.*al*. [26] where, after rainfall, 89% of faunal abundance comprised of *Ae*. *camptorhynchus* (56%) and ostracods (33%), increasing to 91% in drying conditions (*Ae*. *camptorhynchus* 46% and ostracods 45%) [28]. Examination of the aquatic community strongly indicates that the ostracod, *Diacypris* spp. (Crustacea:Ostracoda) [25], is the only plausible competitor for resources with *Ae*. *camptorhynchus* and was, therefore, used in the competition experiment. Mosquitoes and ostracods were collected either after substantial rainfall or tidal inundations that submerged most of the salt marsh which provided newly hatched *Ae*. *camptorhynchus* larvae (1^st^ instars, < 24 hours old). While every attempt was made to collect early first mosquito instars, age was difficult to control in practice, so for consistency examination of developmental rates in this study are restricted to the second instar onwards. All invertebrates were collected using a 350 mL plastic larval dipper (Australian Entomological Supplies Pty. Ltd.).

### Laboratory conditions

Following field collections, mosquito larvae were placed into 500 mL translucent cylindrical plastic containers with 200 mL of water at 35 ppt salinity (“Red Sea Salts”; at 35 ppt elements are 8.2–8.4 pH, 7.8–8.3 Alk (dKH), 420–440 Ca (mg/L), 1250–1310 Mg (mg/L) and 380–400 K (mg/L)), with containers placed randomly in temperature cabinets (Andrew Thorn Limited Qualtex 68 R4) at 23°C ± 0.05 S.E., 14:10 day/night. The temperature and salinity were chosen for laboratory conditions based on the average summer daily temperatures (°C) and salinity (ppt) of water bodies in the field near Hobart [27].

It is difficult to know the exact nutrient variables most relevant to mosquitoes within salt marshes and this is an important area for future studies. As a consequence, invertebrate food consisted of ground “Nutrafin Max Fish Flakes” (Pets Domain). Four grams were ground using a mortar and pestle and suspended in 1 L distilled water. At each feed the solution was agitated to allow for homogeneity and refrigerated at 6°C between feeds to standardise the potential growth of microbes. Although not entirely analogous to their field based diets, food levels and type were representative of other laboratory studies of culicid nutrition and development [29–31] and helped standardise nutritional quality and quantity which could otherwise vary if using field collected resources. New food was prepared fortnightly.

### Experimental design

We conducted a paired experimental design to examine the effects of both intra-specific resource limitation and non-culicid exploitative competition on mosquito development and survival. While a fully crossed design would have been optimal, this was beyond the scope of the study owing to the logistics of available incubators and number of ostracods required. Instead this study provides a paired design where intra-specific resource limitation and non-culicid controphic exploitative competition experiments are contrasted. In the resource limitation experiment 50 larvae were exposed to one of four food resource levels (0.1 mL, 0.2 mL, 0.4 mL or 0.6 mL food/day), with six replicate cylinders per treatment. For the exploitative competition trials 50 larvae were exposed to one of four treatments of competitor (ostracod) density (0 (control), 150, 300 and 600 ostracods/cylinder). This reflects the observed range of ostracod densities per 350 mL larval dippers (Australian Entomological Supplies) in water bodies at Primrose Sands between 2011 and 2012 (1–2144, *n* = 442) [27]. Food resources remained at a constant level of 0.4 mL/day. Each treatment consisted of 10 replicate cylinders, with the non-culicid exploitation control treatment being comparable to the 0.4 mL/day treatment in the resource experiment. Two replicates from each treatment from both experiments were dispersed evenly among three (resource experiment) or five (non-culicid exploitation experiment) independent incubators (akin to blocks), with the position of replicates randomised within each incubator.

Daily counts of mosquito larvae included the number of larvae, instar of each larva, number of pupae and number and sex of adults. Any mosquito larvae or pupae that died were recorded and removed from the container. All emergent mosquitoes were collected, sexed and their wings removed and mounted using water onto glass slides and sealed with clear nail varnish. The average length of left and right wings were used as a proxy for adult size [32]. These were measured at 25× magnification from the wing tip (excluding the fringe) to the arculus [10, 33] using Las EZ software (Leica Microsystems, Switzerland). Developmental time for instars–pupae in each replicate container was as the day at which 50% of surviving larvae reached the next stage of development.

To account for size variation of ostracods among treatments, a subsample of five ostracods from each controphic resource limiting replicate was removed at the beginning of the experiment and again at the end of the experiment (when the last mosquito emerged or died in each replicate container). Measurements of the carapace, from posterior to anterior, were conducted using an ocular micrometer (0.016mm units per graticule unit) on a Nikon SM2800 dissecting microscope at × 6.3 magnification. All remaining ostracods in each replicate were scored for survival.

## Analyses

In both experiments we evaluated how treatment affected larval developmental times, survival, and adult size. Larval development was determined as developmental time when 50% of surviving larvae had reached the next each instar stage, pupa or adult. Survival was measured as the number of emerging adults and size was based on wing length measurements. An alternative approach to evaluating survival is by Cox hazard models, but we could not reliably identify individual survival in our experiment design, precluding this type of analysis. We used a Bayesian mixed effects modelling approach, with incubator as the random effect, owing to its superior ability to estimate coefficients than non-bayesian approaches, in a mixed effects framework [34]. In each model *Y*_*ij*_ (larval developmental time, survival and adult size) was measured for each replicat cylinder *i* = 1,…, *n*_*j*_ for incubator *j* = 1,…, *k*. The distribution of *Y* among replicates was assumed to have a Gaussian distribution with parameter *π*_*ij*_:

$$Y_{ij}|\;\pi_{\text{ij}}∼\text{Gaussian}(\pi_{\text{ij}}\;)$$

where *π*_*ij*_ is the modelled *Y* of replicate *i* in incubator *j*. We modelled the *Y*, *π*_*ij*_, based on the effects of treatment

$$\pi_{\text{ij}}\;=\;\alpha_{ij}+\;\beta_{ji}x_{\text{i}}$$

where *α* and *β* are the model intercept and slope, respectively, for replicate *i* varying by incubator *j*, and *x* was the assigned experimental treatment (intra-specific resource or non- culicid controphic exploitative resource limiting level) for replicate *i*. An additional fixed effect of sex was included for the model of adult size. Prior distributions for all model parameters in the hierarchy (incubators) were given with the goal of providing conjugate priors that contain little to no influence on the posterior distributions of all the model parameters. We assumed normal prior distributions on slopes, *α*, and intercepts, *β*, with mean *μ* and variance *σ* ^*2*^:

$$\alpha_{j}∼\text{Normal}\;(\mu_{\alpha },\;{\sigma^{2}}_{\alpha }),\;\text{for}\;j\;=\;1,\ldots \;,k$$

$$\beta_{j}∼\text{Normal}\;(\mu_{\beta },\;{\sigma^{2}}_{\beta }),\;\text{for}\;j\;=\;1,\ldots \;,k$$

For the variance parameters, *σ* ^*2*^, we determined and utilized non-informative uniform prior hyper-parameter distributions, specified as *σ* ^*2*^~Uniform (0, 100), which was used across all models. Models were fitted in R (v 3.0.3) [35] using the ‘MCMCglmm’ package [36], with MCMC chains run for 13,000 iterations after a burn-in period of 3,000 iterations, ensuring convergence of model parameters, assessed following Gelman and Hill [34]. We summarized posterior distributions of model coefficients, *β*, by the Bayesian median and 95% credible intervals and MCMC simulated *P*-values.

We assessed consistency of results between the two experiments at the 0.4 mL/day intra-specific resource limiting treatment and the control treatment (no ostracods and same food level) from the non-culicid resource exploitation experiment. This was undertaken for development, survival and adult size using a Bayesian mixed effects model as described above, but with experiments being the fixed effect.

Using a Bayesian mixed effects model as previously described, we also investigated ostracod size (measured through sub-samples) and mortality amongst competition treatments (end count of ostracods) to explore if these factors changed *Ae*. *camptorhynchus* developmental times, survival and size or ostracod development and mortality. This analysis was undertaken for quality control purposes, as changes in these could confound non-culicid exploitative competition treatment effects.

## Results

### Development time

**Fig 1:**
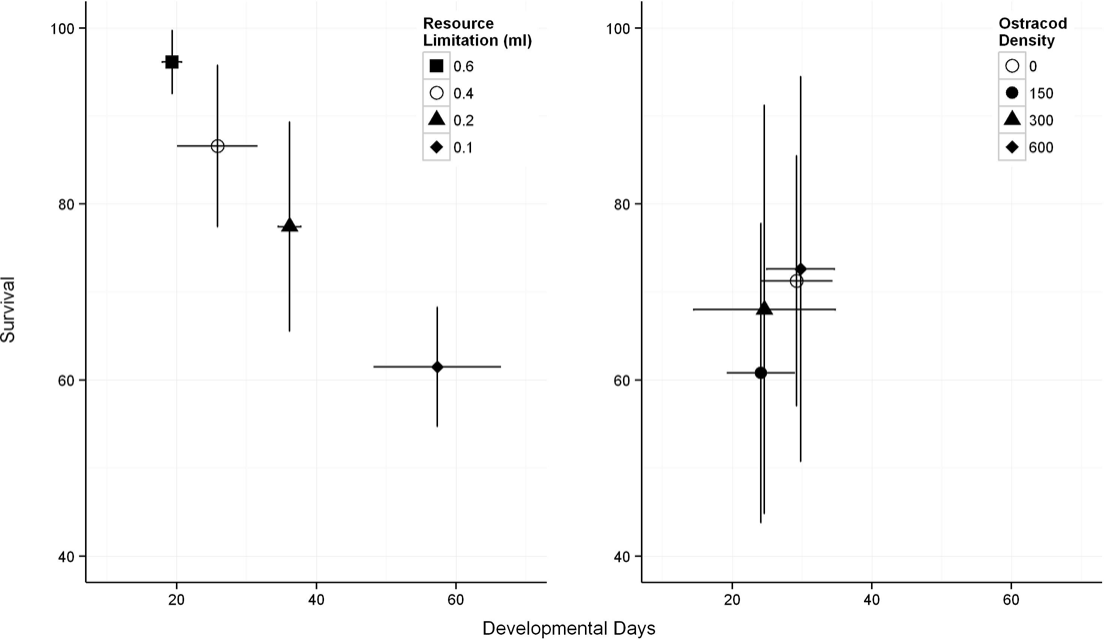
Effects of resource limitation (left panel) and exploitation competition (right panel) on *Ae*. *camptorhynchus* mean development time and survival to adulthood (mean ± SD). Treatments with open symbols are directly comparable.

As the amount of intra-specific resources increased the time it took for *Ae*. *camptorhynchus* to develop decreased (**Table 1**). This relationship was observable within each larval stage with the exception of the pupal stage when mosquitoes do not feed. Overall, the mean developmental time of *Ae*. *camptorhynchus* to adult was 35 days with a range of 21–70 days (**Fig 1**; S1 Table). In contrast, the time taken for *Ae*. *camptorhynchus* larvae to develop was unrelated to the number of competitors. The only significant effect detected was for third instar which developed more slowly with increasing numbers of ostracods (**Table 1**). Mean time to develop to adult was 27 days ranging between of 33–39 days (Fig 1; S1 Table). When comparing the two experiments, there was no difference in developmental time between the 0.4mL/day intra-specific resource limitation treatment and the control treatment (no ostracods and same food level) for the non-culicid exploitation competition experiment (*p =* 0.176) (Fig 1).

**Table 1:** Bayesian mixed-effects regression results showing effects (median coefficient and 95% credible intervals) of both resource limitation and exploitation competition treatments on *Ae*. *camptorhynchus* larval development time (in days) between instar, pupae and total developmental time to adulthood, the effect of treatment on survival and the effect of both treatment and sex on adult wing length (mm) for both resource limitation and competition experiments. Significant *p* values are in bold.

Experiment	Resources				Competition			
	coefficient	2.5%CI	97%CI	p	coefficient	2.5%CI	97%CI	p
	**Development (days)**							
**Treatment effect on larvae**								
**Second instar**	-0.710	-1.169	-0.189	**0.006**	-4.74e-5	-2.15e-3	1.55e-3	0.984
**Third instar**	-3.379	-4.594	-1.789	**<0.001**	0.002	0.260–3	0.003	**0.008**
**Fourth instar**	-1.431	-2.405	-0.293	**0.004**	0.003	-0.002	0.008	0.262
**Pupae**	0.036	-0.137	0.207	0.654	0.002	-0.003	0.008	0.398
**Overall**	-6.893	-8.658	-5.205	**<0.001**	0.003	-0.007	0.013	0.55
	**Survival**							
**Treatment**	11.257	8.088	14.201	**<0.001**	0.006	-0.024	0.036	0.696
	**Wing length (mm)**							
**Treatment**	0.069	0.061	0.077	**<0.01**	<-0.001	<-0.001	<-0.001	**<0.01**
**Sex**	0.038	0.007	0.068	**0.02**	-0.093	-0.037	0.017	0.526

### Survival

*Aedes camptorhynchus* survival decreased as intra-specific resources became more limited (Table 1). The mean range of survival was between 61.4% – 96.1% (Fig 1; S1 Table). By comparison, increasing non-culicid exploitative competition did not affect *Ae*. *camptorhynchus* survival (**Table 1**), with a mean range of survival between 60.8% – 72.6% (**Fig 1**; S1 Table). Comparing the two experiments survival was slightly higher in the resource limitation than the control treatment (no ostracods and same food level) for non-culicid exploitative competition experiments (*p* = 0.028) (**Fig 1**).

### Adult size

**Fig 2:**
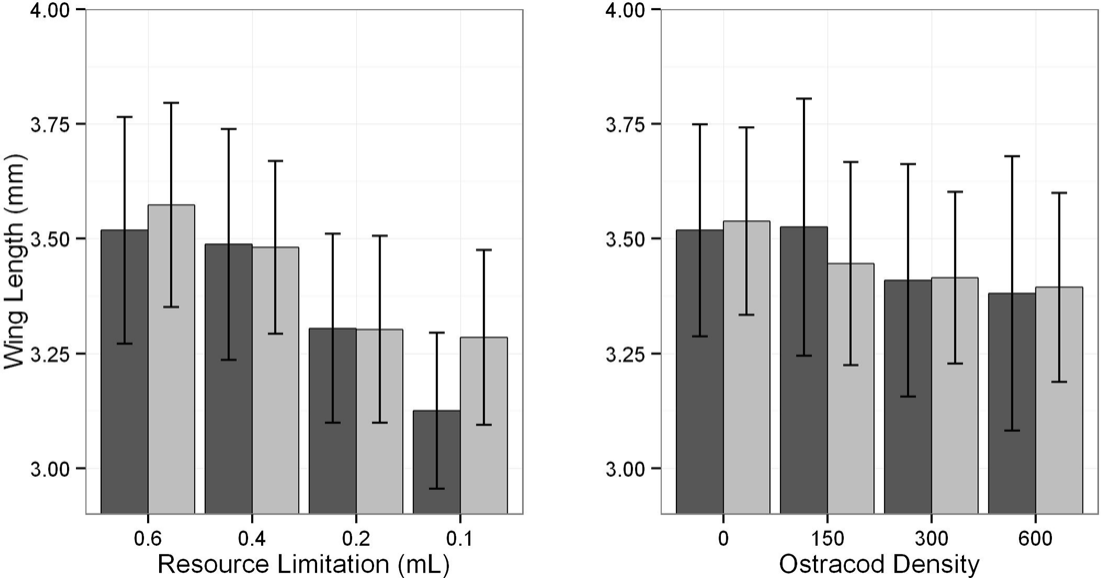
Mean (± SD) wing length (mm) for *Ae*. *camptorhynchus* adult females (dark grey) and males (light grey) for both resource limitation (left panel) and competition (right panel) treatments.

The size of *Ae*. *camptorhynchus* (based on wing length) declined as intra-specific resources became more limiting (**Table 1**). Overall, emerging adults were 9.86% larger in the most resource rich treatment, relative to all the other resource treatments (Fig 2). There was also a sex-specific effect on size with the wings of males being 0.15 mm larger than females, however this was only in the most limiting resource treatment. Likewise, increased non-culicid controphic competitors resulted in decreased size of *Ae*. *camptorhynchus* adults (**Table 1**). On average adults emerging from the control (no ostracods) were 4% larger than adults from the highest ostracod density treatment (**Fig 2**). There was no significant sex-specific size difference between treatments owing to ostracods (**Table 1**). Comparing the two experiments, adult size did not differ (*p =* 0.91, **Fig 2**).

### Ostracod mortality and size

Ostracods within the non-culicid controphic exploitative treatments averaged 0.842 mm in length with no significant difference in size over the duration of the experiment (*P* = 0.624). Similarly, ostracod survival did not significantly differ between experimental treatments (*P* = 0.628), with an overall average 17% loss of ostracods.

## Discussion

Like resource limitation, competitive interactions can affect mosquito life history, thereby potentially having an important role in the ecology of disease vectors. The results of our intra-specific resource limitation experiments are consistent with previous work that has investigated the effects of resource limitation on mosquito development and survival [31, 37–40] showing decreased developmental times, decreased survival and reduced adult sizes as resources become more scarce. However, when numbers of ostracod controphic competitors are increased, at least in our experimental conditions, exploitative competition had no impact on larval *Ae*. *camptorhynchus* developmental traits, but do effect adult size. Adult body size decreased as the density of ostracods increased, suggesting exploitative competition among *Ae*. *camptorhynchus* and ostracods may cause some effects similar to resource limitation. Our study is the first to directly test non-culicid controphic competition with ostracods, which form the same functional feeding group as *Ae*. *camptorhynchus*. We demonstrate that this interaction is an important component to mosquito ecology providing insight into the complexity of aquatic interactions with an important vector species.

As expected, reducing the resources available to *Ae*. *camptorhynchus* drove increased developmental times [31, 38–41] likely through intra-specific competition. Similar extension of developmental time has been demonstrated for both *Ae*. *albopictus* (Skuse) and *Ae*. *aegypti* in limiting diets and this was inferred to indicate impacts on accumulation, assimilation and storage of energy gained from food resources [42]. Prolonged larval developmental times could have negative impacts in the field, especially in conditions where water bodies are extremely ephemeral such as salt marshes. For example, extended developmental durations in these aquatic habitats increase exposure to habitat loss through water bodies drying up [43], thereby having the potential to result in decreased population sizes and reduced disease transmission.

The effects on non-culicid controphic competition on mosquitoes is a relatively new field of research, with few studies detailing increased developmental times in the presence of such exploitative competitors [1, 4, 44]. In these situations, where resources are shared [1, 3, 24], it is common to expect exploitation competition of one species over the other [45, 46]. It was expected that our non-culicid exploitative experiment would show that increasing ostracod density would cause longer *Ae*. *camptorhynchus* development. This expectation was supported by other studies, such as Stav et al. [44] where the authors presented increased time to metamorphosis of *Culex pipens* (Linnaeus) in the presence of *Daphnia magna* (Straus). In our trials the only significant impact on development we observed was during the third instar, with no effect of ostracod density on overall developmental times. While it is plausible that the densities within our trials were inadequate to result in exploitative competitive outcomes on *Ae*. *camptorhynchus* life history, the number of ostracods used in these trials reflect ostracod densities found in natural water bodies [26, 27]. Therefore, given that *Ae*. *camptorhynchus* maintained consistent rates of development across density treatments of ostracods, and ostracod mortality was not significant across treatments, we suggest exploitative competition is unlikely to be limiting larval development in our system.

Similarly, our intra-specific resource limiting trials showed *Ae*. *camptorhynchus* survival declined as resources became limited, but *Ae*. *camptorhynchus* survival did not change with increasing densities of ostracod competitors. Declines in mosquito survival has been documented in other non-culicid controphic competitive studies [1, 13], for example, Mokany and Shine [13] demonstrated a decline in *Aedes australis* survival as a result of interference competition between tadpoles (*Limnodynastes peronii*), however this was a result of chemical interference not exploitative competition. In contrast, Daugherty and Juliano [47] demonstrated improved survival of *Ae*. *triseriatus* in the presence of higher densities of scirtid beetles, because of the quantities of faeces excreted by the scirtids supported microorganisms that nourished the developing mosquito larvae. This, again, is not exploitative competition. Overall, our results suggest that, like developmental rates, the density of ostracods has little impact on mosquito survival through exploitative competition in this system.

Given the lack of detectable effects of ostracod density on *Ae*. *camptorhynchus* larval development and survival, it was somewhat surprising to see an effect on adult size. Limiting resources is known to cause emerging mosquitoes to be smaller [31]. For example, *Ae*. *agypti* adults emerging from nutrient deprived crowded conditions were significantly smaller. Here we suggest ostracod competitors may have a similar effect. Therefore, detecting the effects of competition between ostracods and *Ae*. *camptorhynchus* may be most sensitively measured through adult size rather than larval development and survival. On the other hand, it is possible that there is a coexistence between *Ae*. *camptorhynchus* and ostracods in these habitats and consequentially partitioning of resources driven by differences in species’ niches [45] and that the changes in adult size observed in these trials were driven by intra-specific competition, although further investigation is necessary.

Even though adult size was negatively affected by intra-specific resource limitation, males were significantly larger than females for the most limiting resource treatment. It is known that larval females take longer to develop as a trade-off for greater accumulation of resources which ultimately results in larger sizes and improved fecundity [48, 49]. However, under increased non-culicid exploitative resource limitation, there may be an effect on female *Ae*. *camptorhynchus* reproductive success. In fact, resource limitation is correlated with fewer oogenesis cycles resulting in reduced fecundity [50] and a decreased ability of mosquitoes to carry and transmit disease [8, 31, 41, 51]. Due to *Ae*. *camptorhynchus* being anautogenous [52] thereby requiring a blood meal to complete oogenesis, it is likely that this reduction in size for *Ae*. *camptorhynchus* limits vectorial abilities, although further research is necessary to understand the connection with *Ae*. *camptorhynchus* size and the ability to transmit RRV. A potential caveat is that experimental adults would not represent field adults, however, we have found substantial overlap in mean wing length between field caught adults and experimental adults (competition, 3.46 ±0.27, resources, 3.37 ± 0.26 and field, 3.60 ± 0.44).

Overall our experiments suggest exploitative competition between *Ae*. *camptorhynchus* and ostracods is limited in laboratory conditions. It is possible that environmental conditions may be more important than competition alone in that competitive effects may change given different abiotic conditions [2, 53, 54]. Such changes can be observed when habitats are drying out or with the addition of new invertebrates through rainfall or tide [55]. For example, hatching of first instar larvae may differ to ostracods hatching from dormancy [56, 57]. Such a situation could result in a time window in which first instar mosquitoes have a competitive advantage both in size and nutrient acquisition. It might also be possible that different life stages of *Ae*. *camptorhynchus* larvae are more sensitive to environmental changes or intra-specific competition [58]. Therefore, replicating these experiments in natural conditions in the field, with the addition of a fully crossed design (where all levels of resource limitation are also tested with ostracod densities against *Ae*. *camptorhynchus* development, survival and size) would benefit our understanding on the relationship between *Ae*. *camptorhynchus* inter- and intra-specific interactions and provide further insight on the complexity of competitive systems.

A limitation of our study is that we did not measure adult survival. Epidemiological models of mosquito-borne disease transmission incorporate adult longevity which can have significant effects on disease transmission [59]. However, environmental effects on larval development and survival have been demonstrated to be both condition- and species-specific, with strong associations between adult longevity, developmental times and body size demonstrated for some species [59, 60]. While resource limitation had a greater impact on *Ae*. *camptorhynchus* survival and development, it is possible that densities of non-culicid competitors (and resource limitation) may result in reduced adult longevity especially considering both treatments had an effect on adult size.

We demonstrate that intra-specific resource limitation and controphic competition have a direct impact on adult sizes, however, changes to *Ae*. *camptorhynchus* life history in exploitation competition environments is not so obvious. We conclude that controphic competition, although quite complex, may have a role in influencing vector-borne disease and implications to human health.

## Supporting Information

S1 Table Mean and maximum developmental time and survival for *Ae*. *camptorhynchus* larvae by treatment for both resource limitation and competition experiments. (PDF)
