# Supplementary material for: Resource Limitation, Controphic Ostracod Density and Larval Mosquito Development
Source: PLoS One. 2015 Nov 11;10(11):e0142472. doi: 10.1371/journal.pone.0142472 (PMC4641740; doi:10.1371/journal.pone.0142472)
Supplement: S1 Table — (PDF) [file pone.0142472.s001.pdf]

**Treatment**	**Development**		**Survival**	
**Competition**	**Mean**	**Max**	**Mean**	**Max**
**0**	29.2	37	71.3	100
**150**	24.1	33	60.8	92
**300**	24.6	38	61.2	80
**600**	29.8	39	72.6	84.2
**Resource Limitation**	**Mean**	**Max**	**Mean**	**Max**
**0.6**	19.3	21	96.1	100
**0.4**	25.8	37	86.6	100
**0.2**	36.2	37	77.4	91.7
**0.1**	57.3	70	61.5	68.4
